# Role of traditional beliefs in the knowledge and perceptions of mental health and illness amongst rural-dwelling women in western Nigeria

**DOI:** 10.4102/phcfm.v14i1.3547

**Published:** 2022-09-29

**Authors:** Ifeoma P. Okafor, Damilola V. Oyewale, Chidumga Ohazurike, Adedoyin O. Ogunyemi

**Affiliations:** 1 Department of Community Health and Primary Care, Faculty of Clinical Sciences, College of Medicine, University of Lagos, Mushin, Nigeria; 2 Department of Community Health, Faculty of Clinical Sciences, Lagos University Teaching Hospital, Mushin, Nigeria

**Keywords:** mental health, knowledge, stigma, rural, Nigeria

## Abstract

**Background:**

Globally, the public health importance of mental health has gained significant attention in recent years. In Africa, many traditional belief systems impact the perceptions, attitude and management of mental illness. Women are usually the primary caregivers of mentally ill persons, but they have lower mental health literacy.

**Aim:**

To assess rural women’s knowledge, perceptions and attitudes regarding mental illnesses and the role of traditional beliefs in their management.

**Setting:**

Epe Local Government Area of Lagos State, Nigeria.

**Methods:**

This was a cross-sectional study with a total of 295 rural women recruited through a multistage sampling method. A pretested interviewer-administered questionnaire was used to collect data. Summary and inferential statistics were measured using Epi Info version 7. The level of significance was predetermined at 5%.

**Results:**

A total of 253 questionnaires were adequately filled and analysed. Overall, just over one-third (35%) of respondents had good knowledge and only 26% had positive attitudes towards mental health and illness. About 45% reported that mental illness should first be treated in ‘the traditional way’, whilst 47% felt that there was no need for collaboration between orthodox and unorthodox healthcare for mental illness. Sociodemographic variables were significantly associated with knowledge (educational level *p* = 0.001) and attitude (marital status *p* = 0.001 and ethnicity *p* = 0.001).

**Conclusion:**

Respondents had poor knowledge of and attitude towards mental health, and traditional beliefs played a role in their perception and management of mental illness. We recommend community-based health education programmes to improve knowledge and help-seeking for mental illness amongst rural women.

## Introduction

Mental health includes the emotional, psychological and social well-being of an individual. It affects how the individual thinks, feels and acts. It is a state of well-being in which the individual realises his or her own abilities, copes with the normal stresses of life, works productively and fruitfully and is able to make a contribution to his or her community.^1^ Mental health also includes the ability to enjoy life – to attain balance between life’s activities and efforts made to achieve psychological resilience.

Mental disorders constitute a major cause of disabilities globally, accounting for 7.4% of disability-adjusted life years (DALYs) and 22.7% of all years lived with disability (YLD), according to the Lancet study on Global Burden of Disease Study (2010).^2,3^

In the United States, nearly one in five adults aged 18 years and above has a mental illness, and an estimated 5.2% of the adult population suffers from serious mental illness (SMI).^4^ In England, one in six adults meets the criteria for a common mental disorder.^5^ In Nigeria, 12.1% of adults have a lifetime rate of at least one Diagnostic and Statistical Manual of Mental Disorders, Fourth Edition (DSM-IV) disorder, and 5.8% have a 12-month history of mental disorders.^6^ These estimates draw attention to the public health importance of mental disorders. However, whilst more than 80.0% of the world’s population live in low- and middle-income countries (LMICs) like Nigeria, less than 10% of the affected population have access to appropriate mental health treatment and care.^7^ The causes of mental illnesses are complex in general, and they vary depending on the particular disorder and the affected individual. In most cases, the cause may be unknown but various factors such as biological, psychological and environmental factors may play a role. Therefore, the development of mental disorders is usually attributed to several factors, rather than just one factor.

Studies have shown that there is relatively poor knowledge about mental health and its associated illnesses.^8,9^ The ability to recognise mental disorders is a precursor for proper health-seeking. It is suggested that knowledge of causes, prevention, efficient treatment, early recognition, help-seeking and employment are significant in lessening the stigma and improving attitudes and behaviours related to mental health, hence reducing morbidity and mortality.^10,11,12^ The stigma attached to mental illnesses (especially those rooted in traditional beliefs) prevents affected individuals from coming forward and seeking or receiving the adequate care that they need out of fear of rejection – even by relatives, who may hide them so as to avoid the so-called ‘embarrassment’ or abandon them altogether.^13,14^ Therefore, improving public knowledge about mental health and illness will foster social inclusion of victims.

A study on social distance towards people with mental illness in southwestern Nigeria reported that of 2078 respondents who participated in the study, the level of social distance was seen to increase with the level of intimacy required in the relationship, with 14.5, 24.6 and 60.9% of respondents categorised as having low, moderate and high levels of social distance towards the mentally ill, respectively.^15^ Another community-based study in Nigeria also reported the presence of negative attitudes towards mentally ill persons (MIPs).^16^ This strengthens the evidence of a high level of social distance and stigmatisation of the mentally ill in the country.

Globally, the attitude towards mental illness assumes a similar pattern. Studies carried out in Europe and America have also documented a negative attitude towards mental health issues, even amongst mental health professionals.^17,18^ These poor attitudes amongst lay people and mental health professionals entrench stigma and discrimination and form an important obstacle to accessing care and treatment opportunities across different countries and cultures.^19^

Across Africa, beliefs in supernatural causes of mental illnesses are widely held.^20^ Traditional beliefs in supernatural causes and remedies of mental illnesses influence people’s knowledge and attitudes. Many traditional belief systems in Africa attribute mental health problems to the influence of ancestors or bewitchment, and traditional healers are therefore viewed as the experts in these matters. These traditional healers are consulted as either the first step in the pathway to biomedical mental healthcare or the sole providers of mental healthcare.^7,16,21^

The poor knowledge and attitude towards mental illness exhibited by the families and caregivers of MIPs and society in general leads to poor care-seeking behaviour for those affected.^22^ This course of action, in more cases than not, further worsens their conditions instead of making them better.^23^ Consequently, in many cases, they present to healthcare facilities only when they are too far gone and their mental health too far deteriorated, with nothing much to be done to salvage the situation.

Stigma against MIPs is higher in rural areas.^24^ Women also usually bear a disproportionate burden of caregiving but have lower mental health literacy.^25^ This study assessed the knowledge, perceptions and attitudes of rural women towards mental illness. In addition, we sought to elucidate the role of traditional beliefs in mental health and illness from them. By so doing, we are optimistic about increasing the awareness of mental health and illness as well as detecting areas where interventions can be put in place through education and other public health measures to reduce the burden and exclusion associated with the disease.

## Methods

### Study design

This cross-sectional study was carried out in Epe Local Government Area (LGA), one of the four LGAs in Lagos State.^26^

### Study setting

Epe LGA has 146 towns and villages. As of the 2006 Census, the population of Epe was 181 734, spread over an area of 1185 km.^27^ The population of residents in Epe LGA was projected at 429 706 in 2015, with women making up about half of the population.^28^ It is a traditional settlement of the Ijebu people, who are a subgroup of the Yorubas. Because of migration and commerce, other tribes can be found in Epe. The women in Epe usually engage in petty trading. There are 17 primary health care centres (PHCs) and three secondary healthcare institutions in Epe LGA. Only a few of the PHCs offer mental health services.

### Study population and sampling strategy

This study was conducted amongst adult women who were residents of Epe. Women who had been diagnosed with mental illness and women who were temporary visitors to the study area were excluded from the study. The sample size was calculated using the Cochran formula for descriptive studies^29^:


$$n=z^{2}\text{pq}/d^{2}$$ [Eqn 1]

A sample size of 246 was estimated based on the following parameters: *p* = 0.20, representing the proportion of respondents (20%) who would ‘definitely be disturbed about working on the same job with someone with mental illness’ in a previous study^15^; 95% confidence interval (CI) (corresponding to *z* = 1.96); and power of 80%. To increase precision and make up for incompletely filled questionnaires, nonresponses or cases of dropout, the sample was increased by 20%, thus giving a total of 295 participants.

A probability sampling in multiple (five) stages was employed for the study. Stage 1 was the selection of wards. Epe LGA has a total of 19 wards. The study was carried out in four of the wards selected by a simple random sampling method. The selected wards were Ajaganabe, Popo-Oba, Oke-Balogun and Odomola. In stage 2, five streets each were selected from three wards (streets in the fourth ward were not well defined) by simple random sampling to give a total of 15 streets out of a total of 40 streets in the three wards. In stage 3, houses were selected. A total of 73 questionnaires were administered in each ward (295/4 = 73). In the wards with well-defined streets, there were about 25–28 houses on each of the selected streets. Houses were then selected by simple random sampling (ballot), that is, 14 houses per street (73/5 = 14). In the ward in which the streets were not well defined, the first 73 houses meeting the inclusion criteria of being residential houses with at least one adult woman were selected. Houses were skipped if they were nonresidential, such as churches, mosques, shops and schools. In stage 4, households were selected. A household represents people who live together and eat from the same pot. Where there was more than one household in a house, one of them was selected by simple random sampling (ballot). In stage 5, respondents were selected. Where there was more than one eligible respondent in a household, one of them was selected by the simple random sampling (ballot) for interview.

### Data collection

A pretested interviewer-administered questionnaire was used as the tool to generate quantitative data. Face validation was performed by experts at the University of Lagos to confirm that it captured the topic effectively, and a high face validity was found. Data collection was conducted by five interviewers, the principal researcher and four research assistants. A pretest was performed amongst 20 residents of Ikorodu LGA, which shares similar demographic and socio-economic characteristics as the study area. This helped to eliminate ambiguous questions before actual data collection. The respondents were interviewed over a period of one month. They were interviewed in their homes for information on the following: sociodemography, basic information about mental health and illness such as causes, symptoms and treatment and caring for one’s mental health, amongst others. Next, they were questioned regarding their perceptions of and attitudes towards mental illness: ‘what would you do when someone starts showing signs of mental illness?’ Likert statements were used on their perceptions of mentally ill people, to which the respondents agreed or disagreed, such as ‘they could infect or affect me’, ‘they should be treated with caution’, ‘they are being treated unfairly’, and so on. Lastly, a series of statements on a Likert scale were used to assess respondents’ traditional beliefs regarding mental health and health-seeking for mental illness. Some of them are as follows: ‘mental illnesses are mainly caused owing to spiritual reasons’; ‘MIPs should first be treated by the traditional way’; ‘traditional medicine is more effective in treating MIPs than modern medicine’; ‘traditionalists and western doctors do not do the same job and should not work together’; and so on.

### Data analysis

Data were analysed using the Epi Info Statistical Package version 7. From the questionnaire, eight questions assessed respondents’ knowledge of mental health, whilst 10 questions assessed their attitudes to and perceptions of mental health. To have an overall assessment of the level of knowledge and attitude, a scoring and grading system was used. A correct response to a knowledge question was scored 1, whilst a wrong response was scored 0. The minimum score for knowledge was 0 and the maximum score was 16 points. This was further graded as follows: 0–7 for poor knowledge and 8–16 for good knowledge. Attitudes and perceptions were assessed on a three-point Likert scale. This was scored by assigning 1 point for disagree, 2 points for neutral and 3 points for agree. For the attitude scale, the minimum score was 10 points whilst the maximum was 30 points, and it was further graded as follows: 8–18 for poor attitude and 19–30 for good attitude.

Data were summarised using means and standard deviations (s.d.). Chi-square was used to test for associations between categorical variables. Statistical significance was set at a *p*-value of 0.05.

### Ethical considerations

Ethical approval (reference number ADM/DCST/HREC/APP/064) was obtained from the Health Research Ethics Committee (HREC) of the Lagos University Teaching Hospital. Written informed consent was obtained from the participants prior to recruitment. Privacy and confidentiality were maintained, and the study participants had the option to withdraw from the study at any time should they wish so.

## Results

A total of 253 questionnaires were adequately filled and analysed, and this was above the minimum calculated sample size of 246.

About half of the respondents were less than 35 years of age (52.18%), with a mean age of 37.11 years and an s.d. of ± 13.84 years. Married respondents, Muslims, those belonging to the Yoruba tribe and respondents with at least a secondary school certificate constituted about 65.61, 50.59, 67.98 and 69.96%, respectively (Table 1).

**TABLE 1:** Sociodemographic characteristics of the respondents.

Variables	Frequency (*n* = 253)	%
**Age (years)†**		
˂ 35	132	52.18
35–55	90	35.57
˃ 55	31	12.25
**Marital status**		
Single	66	26.09
Ever married	187	73.91
**Religion**		
Christianity	123	48.62
Islam	128	50.59
Others	2	0.79
**Educational qualification**		
No formal education	30	11.86
Primary school	46	18.18
Secondary school	94	37.15
Tertiary	83	32.81
**Ethnicity**		
Yoruba	172	67.98
Hausa	10	3.95
Igbo	32	12.65
Other	39	15.42
**Employment status**		
Employed	185	73.12
Unemployed	68	26.88

† , Mean ± s.d. = 37.11 ± 13.84.

About two-fifths of respondents did not know the meaning of mental illness (41.90%), and the majority could identify the causes of mental illness (90.90%), of which 60.87% identified personal factors such as stress, worry, loss of a loved one and substance abuse as causes. The majority of respondents knew the symptoms of mental illnesses (93.66%), of which 78.90% mostly knew unusual behaviour (Table 2).

**TABLE 2:** Respondents’ knowledge of mental health and illness.

Statement (*n* = 253)	Frequency	%
**Meaning of mental health**		
Being mentally sound	76	30.04
The absence of mental illnesses	37	14.62
Both	34	13.44
I don’t know	106	41.90
**Causes of mental illnesses** †		
Biological factors	114	49.57
Personal factors	154	60.87
Spiritual factors	118	51.30
I don’t know	22	8.70
Others	1	0.40
**Symptoms of mental illnesses** †		
Withdrawal	44	18.57
Worry	35	14.77
Changes in weight or appetite	29	12.24
Depression	48	20.25
Emotional outbursts	49	20.68
Strange thinking	86	36.29
Difficulty sleeping	39	16.46
Unusual behaviour	187	78.90
Aggression	64	27.00
I don’t know	14	5.53
Others	2	0.79
**Meaning of mental healthcare** †		
Prevention of mental health problems	92	55.09
Treatment of mental health problems	110	65.87
Observing mental hygiene	69	41.32
I don’t know	86	33.99
Knows places to receive mental healthcare	130	51.38
**Places to receive mental healthcare† (*n* = 130)**		
Mental healthcare centres	51	39.23
Hospital	82	63.08
Home	6	2.37
I don’t know	3	1.19
Others	2	0.79
**Treatment of mental illness** †		
Use of drugs	47	20.17
Seeing any doctor	47	20.17
Seeing a psychiatrist	40	15.81
Seeing a spiritualist	156	66.95
There is no treatment	5	2.10
I don’t know	20	7.91
**Overall knowledge**		
Good	89	35.18
Poor	164	64.82

† , Multiple responses.

Most of the respondents (65.61%) agreed that ‘mental health is important’; 39.13% admitted that they would not relate with an MIP, whilst almost half of the respondents (47.82%) reported that they would keep their distance from such a person (Table 3).

**TABLE 3:** Respondents’ attitude towards mental health.

Statement (*n* = 253)	Agree (*n*)	%	Neutral (*n*)	%	Disagree (*n*)	%
Mental health is important	166	65.61	79	31.23	8	3.16
I can keep relationships with the mentally ill	64	25.30	90	35.57	99	39.13
They are not dangerous	18	7.11	182	71.94	53	20.95
They can be trusted	26	10.28	26	10.28	201	79.44
They are stable and predictable	11	4.35	9	3.56	233	92.09
They are easy to cope with	10	3.95	63	24.90	180	71.15
They cannot affect or infect me	102	40.32	79	31.23	72	28.46
They are being treated unfairly	58	22.92	121	47.83	74	29.25
They should be treated like normal people	36	14.23	49	19.37	168	66.40
**What to do if someone shows signs of mental illnesses**						
I would keep my distance	121	47.82	-	-	-	-
I would seek help	93	41.70	-	-	-	-
I would ignore	5	2.24	-	-	-	-
I would wait a while for improvement	35	15.70	-	-	-	-
I don’t know	30	11.86	-	-	-	-
**Overall grade of attitude**						
Positive	67	26.48	-	-	-	-
Negative	186	73.52	-	-	-	-

Most respondents (63.24%) believed that evil spirits could cause mental illnesses; 60.87% believed that curses from supernatural beings (gods) could lead to mental illnesses, whilst 41.91% believed that the influence of one’s ancestors, regarding past sins, could lead to mental health problems. However, 46.64% of the respondents disagreed that the head of the household should be the one making decisions regarding mental health for the rest of the household. Over one-third of the respondents (36.50%) believed that persons suffering from mental illness should be hidden. About 45.00% believed that the ‘traditional’ way is in fact the first path that should be taken in the treatment of people with mental illness, whilst about one-third agreed that traditionalists were better than western doctors in the treatment of mental illness. Similarly, 34.47% of participants agreed with the statement that ‘traditional medicine is more effective for treating mental illness than modern medicine’. More than one-third (35.79%) reported that they shouldn’t be associating with mentally ill people. Almost half (47.31%) felt that there was no need for collaboration between medical doctors and traditionalists with regard to mental health (Table 4).

**TABLE 4:** The role of traditional beliefs in mental health.

Statement	Agree (*n*)	%	Neutral (*n*)	%	Disagree (*n*)	%
Evil spirits cause mental illness	160	63.24	45	17.79	48	18.97
Supernatural curses can cause mental illnesses	154	60.87	54	21.34	45	17.79
Ancestral influences as a result of past sins can cause mental illnesses	106	41.91	77	30.38	70	27.71
The head of the household should make decisions regarding mental health	75	29.74	60	23.72	118	46.64
Mentally ill people should be hidden	92	36.50	79	31.06	82	32.44
Mentally ill people should be killed	26	10.28	64	25.51	16	64.21
Mentally ill people should first be treated in the ‘traditional’ way	113	44.61	60	23.72	80	31.77
Traditionalists are better at treating mentally ill people than western doctors	80	31.77	94	37.14	79	31.09
Traditional medicine is more effective in treating mental illnesses than modern medicine	87	34.47	91	35.79	75	29.74
Tradition demands that I should not associate myself with mentally ill people	91	35.79	94	37.17	68	27.04
Regarding mental health, traditionalists and western doctors do not do the same job, and therefore, no need for collaboration	120	47.31	89	35.12	44	17.57

There was a statistically significant association (*p* < 0.001) between educational level and knowledge about mental illness, as those with tertiary education had better knowledge.

Married respondents had more negative attitudes towards mental illness, whilst those who were not of the Yoruba ethnic group had more positive attitudes, and these differences were statistically significant (*p* < 0.001 and *p* < 0.001, respectively) (Table 5).

**TABLE 5a:** Sociodemographic factors associated with knowledge.

Variable	Poor knowledge (*n*)	%	Good knowledge (*n*)	%	*χ* ^2^	*p*
**Age (years)**						
˂ 35	92	69.70	40	30.30	-	-
35–55	53	58.89	37	41.11	-	-
< 55	19	61.29	12	38.71	2.94	0.231
**Religion**						
Christians	86	69.92	37	30.08	-	-
Non-Christians	78	60.00	52	40.00	2.73	0.099
**Educational level**						
No formal education	24	80.00	6	20.00	-	-
Primary school	40	86.96	6	13.04	-	-
Secondary school	65	64.15	29	30.85	-	-
Tertiary institution	35	42.17	48	57.83	32.37	< 0.001
**Ethnicity**						
Yoruba	112	65.12	60	34.88	-	-
Non-Yoruba	52	64.20	29	35.80	0.02	0.886

**TABLE 5b:** Sociodemographic factors associated with attitude.

Variable	Negative attitude (*n*)	%	Positive attitude (*n*)	%	*χ* ^2^	*p*
**Marital status**						
Single	41	62.12	25	37.88	-	-
Married	135	81.33	31	18.67	-	-
Divorced/widowed	10	47.62	11	52.38	16.84	0.001
**Religion**						
Christians	90	73.17	33	26.83	-	-
Non-Christians	96	73.85	34	26.15	0.01	0.903
**Educational level**						
No formal education	20	66.67	10	33.33	-	-
Primary school	42	91.30	4	8.70	-	-
Secondary school	70	74.47	24	25.53	-	-
Tertiary institution	54	65.06	29	34.94	11.29	0.103
**Ethnicity**						
Yoruba	139	80.81	33	19.19	-	-
Non-Yoruba	47	58.02	34	41.98	14.69	0.001

## Discussion

Overall, the majority of respondents had poor knowledge of and negative attitude towards mental health. They also mostly agreed that traditional beliefs, spiritual factors such as curses, karma and witchcraft had a significant role to play in mental health and illness.

Our respondents mostly attributed mental illness to personal and spiritual factors, with unusual behaviour being the most common symptom mentioned. The majority of them described mental healthcare as treatment of mental illnesses. This portrays a deficit in knowledge, implying that most would present only when a problem arose. Many rural residents in Africa also viewed mental disorders as originating from personal factors such as alcoholism, the spiritual realm (such as bewitchment),^30^ stress, poverty and God’s punishment.^24^ Urban residents in Yaoundé, Cameroon equally had poor knowledge of the causes of mental illnesses and only about half could properly identify the signs and symptoms associated with it.^31^

Many women knew health facilities as the place to receive mental healthcare, but then went on to say that consulting spiritualist was the main method of treatment. Cameroonians were also reported to have a poor knowledge of treatment of mental illness.^31^ In Zanzibar, community members (90.72%) knew health facilities as places of help-seeking, in addition to traditional (53.98%) and religious (31.12%) healers.^30^ Healthcare professionals have lamented poor community awareness of mental disorders and societal beliefs in traditional healers and prayers as major barriers to appropriate help seeking for mental illness.^32^ Thus, there needs to be increased awareness about mental health and illness, as well as appropriate treatment. Higher educational attainment was significantly associated with good knowledge. In addition, younger respondents (< 35 years) had better knowledge, but the difference was not statistically significant. In Cameroon, younger respondents had significantly better knowledge,^31^ and also in Scotland, it was shown that youth tended to have a good knowledge of mental health and how to go about helping people with mental illnesses.^33^ Higher education comes with more exposure to information, and these days the youth are more enlightened and educated as they have better, easier and faster access to information, especially with the advent of the Internet, social media and the like. Thus, education of the girl-child cannot be over-emphasised.

A majority of the respondents reported that they would keep their distance from an MIP. Many could not keep relationships with MIPs, nor did they agree that they should be treated like normal people. Almost 70% of respondents in Ilorin (Southwest Nigeria) would not mind talking to an MIP, yet two-thirds of them opined that MIPs should not be treated the same way as ‘healthy’ people.^32^ Similar attitudes were also reported by other researchers in Southwest Nigeria^15,34^ and other parts of Africa.^24,31^ This reflects the very high level of stigmatisation against MIPs in society. Marital status and ethnicity were factors that significantly influenced attitudes to mental illness, where widowed or divorced women and those of non-Yoruba tribes had more positive attitudes to mental health and illness. Cultural variations may account for these differences, and again, those who are divorced may have experienced some stigma – hence their positive attitude is a sign of empathy. In Ethiopia, marital status (never married), ethnicity (Oromo) and residence (rural), in addition to educational status (illiterate), were positively associated with stigma against MIPs.^24^ In rural Istanbul, Turkey, women with higher educational and economic backgrounds had more negative attitudes to mental illness, whilst younger and married women had significantly more positive attitude.^35^

The preference for traditional means as the first choice of treatment is not surprising for a number of reasons. The respondents believed in the ‘demonic’ (supernatural) origin of mental illness causation and hence that traditional treatment should be the mainstay. Again, the rural location of the study may have also contributed to this preference. The preference for traditional treatment is common in rural areas,^30^ or a combination of traditional and Western methods.^24,36^ The choice of treatment can depend on the severity, and the fees paid to spiritual healers may be up to 120 times more than hospital or clinic fees.^36^ Mentally ill persons are usually subjected to harmful treatments in these unorthodox centres, where they may be kept in chains, forced into seclusion and made to suffer sexual abuse and other forms of exploitation.^37,38^ Studies have reported a huge gap in the treatment of mental disorders in rural areas and included poverty and stigma as part of the issues that act as barriers to appropriate treatment for MIPs.^32,39^ Another Indian study reported orthodox healthcare as the first choice for mental illness.^40^ This study was a facility-based study, which could have introduced some bias. The respondents also largely disapproved of orthodox and unorthodox collaboration in the management of mental illness. As revealed in a study, orthodox medicine has fallen below expectations of patients and their caregivers, and both groups of practitioners also expressed reservations regarding collaboration.^41^

In general, observations from this study align with those of recent scoping reviews of the knowledge and attitude towards mental illness in Nigeria.^42,43^ The reviews covered institutional and community levels in different parts of the country. They showed that Nigerians commonly ascribed mental illness to supernatural causes, expressed stigmatising attitudes towards victims and preferred unorthodox healthcare for management, implying that little has changed over time.

Our findings have shown that poor knowledge, poor attitudes towards mental health and the high perception of strong links between traditional belief and mental illness will impact prevention and control efforts. These are points for intervention to increase community awareness, reduce stigma and improve health-seeking behaviour. With the recent inclusion of mental health in the primary health care system in the country, rural communities should be empowered with necessary information on mental health, as their involvement is considered vital in strengthening the services, as well as other health system reforms for improved appropriateness and quality of service.^44,45^

### Strengths and limitations of the study

This study was conducted with robust methodology in a rural setting where there is usually a dearth of data, especially in the context of LMICs such as Nigeria. It was done amongst women who disproportionately bear the burden of care as the caregivers for MIPs. It also highlights the stigmatisation faced by MIPs in communities.

Whilst this study brings to fore the influence of long-held traditional beliefs with regard to mental illness care and treatment, respondents mostly responded to questions with reference to ‘overt’ mental illness, whilst there are many individuals with ‘covert’ illnesses. A study that includes other segments of population, especially men, may reveal other unique perspectives in the study area. Because of the nature of the topic, qualitative data collection may have been valuable in exploring other traditional and cultural aspects of attitude and practices regarding mental illness. Despite these shortcomings, our study has contributed to the body of knowledge on this topical issue and the findings should spur stakeholders into committed actions.

## Conclusion

Respondents in this study had poor knowledge of mental health and stigmatising attitudes towards people with mental illness. Traditional beliefs played a significant role in their perception of mental health and its health-seeking behaviour. Knowledge was significantly associated with educational qualifications and attitude with the marital status and ethnicities of the respondents. Community-based health education programmes involving custodians of tradition and caregivers should be organised to improve knowledge, dispel myths, reduce stigmatisation and improve health-seeking behaviour for mental illness amongst women in rural communities.
